# Smart Sensor Based on Biofeedback to Measure Child Relaxation in Out-of-Home Care

**DOI:** 10.3390/s20154194

**Published:** 2020-07-28

**Authors:** Daniel Jaramillo-Quintanar, Irving A. Cruz-Albarran, Veronica M. Guzman-Sandoval, Luis A. Morales-Hernandez

**Affiliations:** 1 Mechatronics, Engineering Faculty, Campus San Juan del Rio, University Autonomous of Queretaro, San Juan del Rio, Queretaro 76803, Mexico; djaramillo15@alumnos.uaq.mx (D.J.-Q.); icruz@hspdigital.org (I.A.C.-A.); 2 Psychology/Psychology Faculty, University of Colima, Colima 28040, Mexico; gus_vero@ucol.mx

**Keywords:** children in out-of-home care, thermal biomarkers, biofeedback, relaxation, smart sensor

## Abstract

Children from out-of-home care are a vulnerable population that faces high stress and anxiety levels due to stressful experiences, such as being abused, being raped, and violence. This problem could have negative effects on their bio-psycho-social well-being if they are not provided with comprehensive psychological treatment. Numerous methods have been developed to help them relax, but there are no current approaches for assessing the relaxation level they reach. Based on this, a novel smart sensor that can evaluate the level of relaxation a child experiences is developed in this paper. It evaluates changes in thermal biomarkers (forehead, right and left cheek, chin, and maxillary) and heart rate (HR). Then, through a k-nearest neighbors (K-NN) intelligent classifier, four possible levels of relaxation can be obtained: no-relax, low-relax, relax, and very-relax. Additionally, an application (called i-CARE) for anxiety management, which is based on biofeedback diaphragmatic breathing, guided imagery, and video games, is evaluated. After testing the developed smart sensor, an 89.7% accuracy is obtained. The smart sensor used provides a reliable measurement of relaxation levels and the i-CARE application is effective for anxiety management, both of which are focused on children exposed to out-of-home care conditions.

## 1. Introduction

Children in out-of-home care are a vulnerable group whose members have frequently been abused or raped or have not been provided with the basic needs by their family. Therefore, they are often assigned to a place that aims to improve their wellness [1,2]. Such mistreatment or deficiencies can have negative effects on their bio-psycho-social well-being [3]. Emotions such as anxiety, fear, and sadness that children in out-of-home care feel arise when a child is involved a situation and appraises it as being immediately relevant to a child’s active goals [4]. Anxiety is a diffuse and unpleasant feeling characterized by hyper-reactivity of the sympathetic system, manifested in physiological parameters and facial expressions. In addition, anxiety has been related to mental health [5]. Therefore, a variety of interventions have been carried out to address this problem, focused on physical activity [1,6], psychosocial attributes [7], and mental health [8], among others. Regarding mental health, it has been documented that emotional states can be inferred through facial expressions, which in turn can reflect the mental state of an individual [9]. In order to know the emotional state of subjects, work has been carried out to detect the following emotions: a neutral state, joy, anger, surprise, fear, sadness, and disgust, among others. These investigations have used physiological signals measured with contact sensors [10,11,12,13,14,15]. Additional studies have been carried out to detect emotions using infrared thermography [16,17,18,19]. These studies have generated great interest due to the fact that this technology is non-invasive, cost effective, and insensitive to lighting [20]. Negative emotions are useful to the body; however, they cause damage when they are experienced repeatedly and for prolonged periods of time [21]. The emotional regulation of these negative emotions is a process that includes increasing or decreasing the intensity of emotion experience, expression, or physiological parameters. The emotional regulation can be intrinsic (self-regulation) or extrinsic (interpersonal-regulation). Indeed, problematic emotions appear with hyper-reactivity, which is conceptualized as an overreaction to a situation [4]. Relaxation is used to deal with stress and anxiety, and involves physiological effects that are the opposite to effects caused by psychological stress. In particular, guided imagery [22] and diaphragmatic breathing [23] decrease the Sympathetic Nervous System (SNS) activity, as well as increase parasympathetic nervous system activity. Biofeedback has emerged as a technique which teaches individuals to recognize and modify their body’s physiological signals to help improve their health [24]. Child monitoring of physiological parameters, whilst based on audio-visual signals, has been conducted in order to help children increase, maintain, cope with, or even recover from an emotional state [25]. Among the physiological parameters that can be measured for this technique to work are the heart rate (HR) variability [22,23,24,25], electromyography (EMG) [26], electrocardiogram (ECG) [27], HR [28,29], temperature [26], ultrasound [30], and electroencephalogram (EEG) [31], among others. The main uses of biofeedback are to decrease anxiety and pain sensations [32], induce relaxation [33], reduce chronic fatigue [34], ameliorate stress [35], manage post-traumatic stress disorder [36], and, in general, improve the quality of life [37,38]. Moreover, biofeedback has been used in video games to reduce anxiety and improve skills in children [39]. Indeed, video games that integrate engaging cognitive training with real-time bio-sensing and neuro-stimulation have the potential to optimize cognitive performances in health and disease [40]. Clearly, tools have been developed to identify emotions through innovative and robust methods. However, the actual level of emotion experienced, needed to emotional regulation, has not been quantified. Published studies only indicate the presence or absence of emotions, without assessing the level of intensity required. Furthermore, there are no reports of studies carried out on vulnerable populations. This is rather unfortunate because these populations are those that need these technologies the most. For this reason, it would be highly desirable to have a method to induce relaxation in vulnerable children, as well as a non-invasive smart sensor that provides knowledge of the degree of relaxation that the subject is experiencing.

The current work presents the development of a smart sensor based on thermography and HR measurement to assess relaxation in out-of-home care children. As an incentive, an application called i-CARE is used, which trains the children with biofeedback and recreates a virtual interactive space that helps reduce anxiety, creating a relaxation feeling in a harmless way. The sensor measures changes in six thermal biomarkers (forehead, left and right cheek, nose, chin, and maxilla) and heart rate. To determine the relaxation level, a k-nearest neighbors (K-NN) classifier is used. 

## 2. Materials and Methods

**Figure 1:**
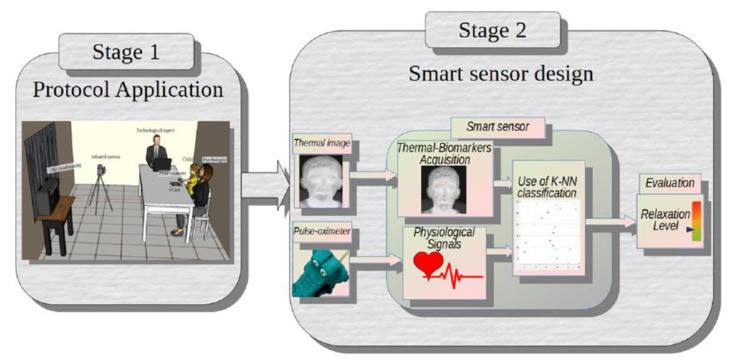
General methodology.

The methodology followed for the development of this system consists of two main stages: (1) the protocol application to induce relaxation; (2) the smart sensor design to assess child relaxation level. The diagram of Figure 1 shows the general methodology followed for the development of this investigation.

### 2.1. Technological Equipment

For the infrared thermogram, an FLIR A310 camera was used, which has a thermal sensitivity of 0.05 at 30 °C, an infrared resolution of 320 × 240 pixels, and a spectral range between 7.5 and 13 µm. This camera was installed on a tripod at a height of 1.2 m and at a distance of 1.2 m from the subject under study. To measure the ambient conditions, a fluke 975 device was used to measure the air quality. Additionally, a fluke 61 laser thermometer was used to measure the reflected temperature.

The HR was measured using a pulse-oximeter designed by Jaramillo-Quintanar [41] that provides measurements of HR and the percentage of oxygenation in the blood every 3 s, which was calibrated with respect to a pulse-oximeter of commercial use endorsed by the Food and Drug Administration (FDA) with an average error of less than 5% [42]. This system was designed for use in pediatric instances, aimed at the acquisition of physiological signals (HR and oxygen saturation) in a child-friendly way.

### 2.2. Conditioned Space

**Figure 2:**
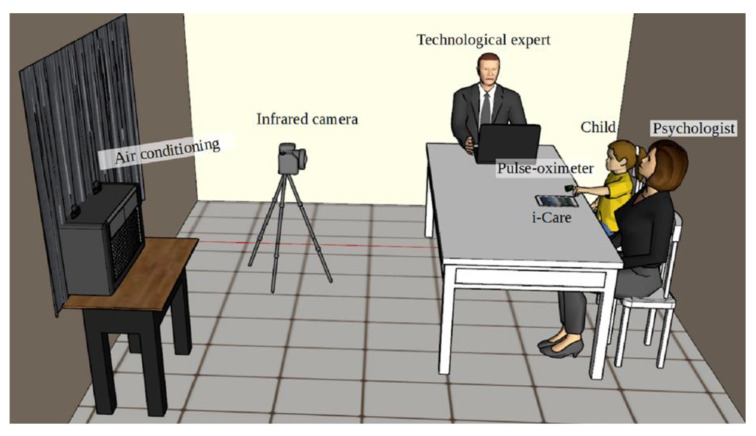
Conditioned space.

To carry out the study, a controlled environment was created (Figure 2) inside a room (2.5 m long, 3 m wide, and 2.5 m high) with constant temperature (20 ± 2 °C), lighting, and relative humidity (45–60%). To achieve this goal, an air conditioning system was used.

### 2.3. Children

The tests were performed on a group of 29 children from an out-of-home care institution, with an average age of 8.7 years and a standard deviation of 1.8. All of the participants had high stress levels due to the following experiences: family problems; economic troubles; institutionalization; diffuse attachments and violence by action; or omission of care. Exclusion criteria consisted of the following: not wearing glasses or accessories in the facial area during infrared thermographic images; being free of any disease that may cause changes in the body temperature; avoiding the use of lotions, creams, cosmetics, and deodorants; not having taken energy drinks; not having exercised an hour before; and finally, their face had to be cleared of hair.

To cover the bioethics criteria, the guidelines of the General Health Law were followed and, in accordance with the Helsinki declaration, the research project was submitted to the Applied Bioethics Committee for Research of the Faculty of Engineering of the Autonomous University of Querétaro (registration key CEAIFI-032-2017-TI). In addition, authorization from the management of the institution and legal guardian of the participants was obtained. The procedures were applied whilst respecting the dignity and privacy of the participants, using informed consent, only applying non-invasive methods, and providing the freedom to participate and leave the intervention when desired.

### 2.4. i-CARE

The i-CARE application was used for training [43]. i-CARE is an application employed for the remote monitoring of physiological variables of anxiety based on the Body Area Network (BAN) paradigm [44] (designed with funding from the 2014–2016 CONACYT National Postdoctoral Stays Program at UNAM). i-CARE ^©^ is a technological training program employed for the management of anxiety; in fact, it involves psychological techniques such as biofeedback, diaphragmatic breathing, guided imagery, and video games. Additionally, i-CARE graphs in real time the data obtained from biosensors for oxygen saturation and HR (sympathetic symptoms of anxiety) and includes a measurement of the subjective parameter of anxiety through the Visual Analog Scale (VAS), as well as a virtual recreational space to create a relaxation effect through video games. Importantly, the system provides biofeedback training for enabling children to learn to regulate the physiological variables of anxiety. This technology allows the acquisition of pertinent physiological data, real-time recording, and averaging of the data for each subject.

#### i-CARE Functions

The i-CARE application has the following functions.

**Figure 3:**
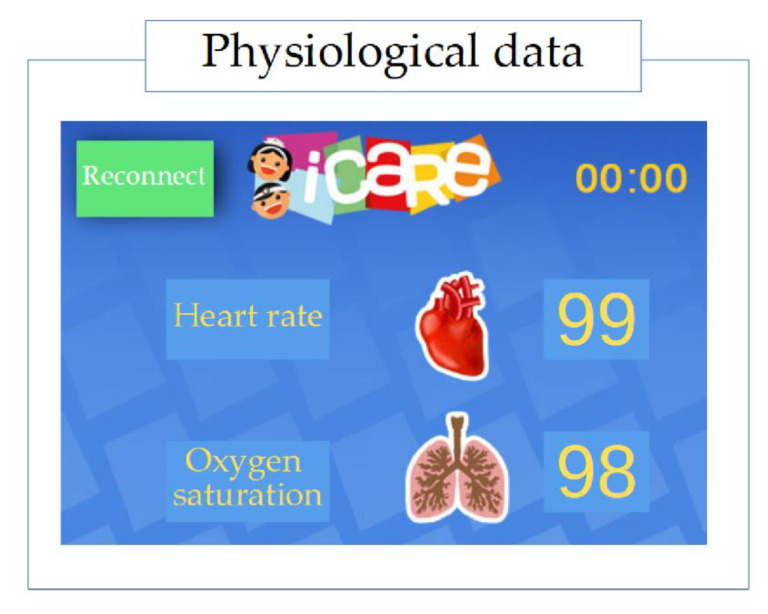
Physiological data shown on i-CARE.

First, the physiological parameters, such as the HR and oxygen saturation, were detected by the sensor on the child’s finger. Then, the signal was displayed on i-CARE’s screen. Finally, every second, the application showed in real time the physiological parameters for the duration of each phase and could be modified by children viewing the animated images (Figure 3). Psychological training by i-CARE is a new way to manage breathing and emotional regulation.

i-CARE involves visual and auditory feedback for the subject regarding the increase or decrease of the measured variable. Each of the colors, shapes, and waves were previously tested through usability studies of the application. Regarding the sounds and melodies incorporated in the application, pleasant, calming music was used [45]. The operation of i-CARE is as follows: 

Registration or research of the subject in an electronic file (sociodemographic data).

Phases of application:

- Phase 1. Baseline (3 min). In this phase, the child remained seated in silence, while their physiological parameters were evaluated by the smart sensor. The instructor (Psychologist) was only an observer;

Training

- Phase 2. Training based on biofeedback (10 min). The instructor trained the child in diaphragmatic breathing and used the physiological parameters for biofeedback (visual signal on i-CARE’s screen in real time);
- Phase 3. Training in relaxation through guided imagery (10 min). On this screen, there was an ocean picture and relaxing music was played ex professo on i-CARE. The instructor trained the child on relaxation through guided imagery (therapeutic narrative), while his physiological parameters were evaluated by the smart sensor and were displayed on i-CARE’s screen;
- Phase 4. Video game (5 min). In this phase, two screens appeared with images of a female doctor, a male doctor, and different clothes, the child could choose the character to play with, and the instructor explained the activity. This phase created a virtual recreational space for the child to relax in and promoted attention, classification, and self-efficacy skills in the child. Physiological parameters were evaluated, but did not appear on the screen for a better visualization of the game.

In every phase, there was the option to skip or save the activity. At the end of i-CARE, averages of the physiological parameters of the four phases appeared on the screen.

### 2.5. Protocol Application

**Figure 4:**
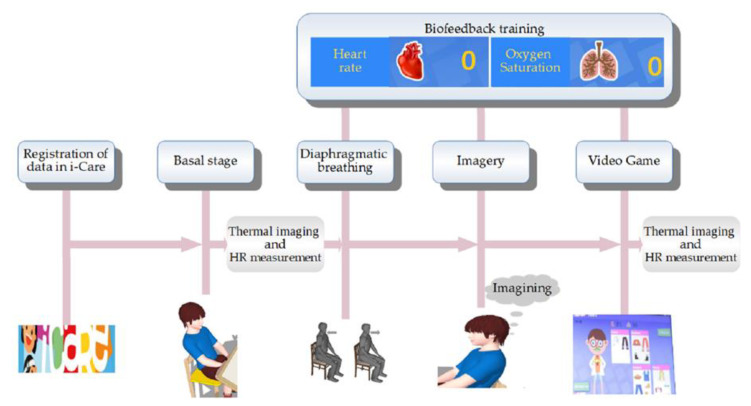
i-CARE protocol.

In each test, the protocol shown in Figure 4 was followed, with the support of a psychologist specialized in the pediatric field, who had uninterruptedly treated these children for a period of more than 1 year, as well as with the collaboration of a specialist in infrared thermography and HR measurements.

This protocol was strictly followed throughout the study, starting with the admission of the child, who was individually admitted to the previously conditioned room detailed in Section 2.2, in which only the current participant in the research and the researchers were present. Prior to admission to the conditioned room, the children were told what the study consists of, and they were made aware of their rights as participants, in addition to signing an informed consent. In addition, an assessment was made to ascertain that no violation of any of the exclusion criteria (addressed in Section 2.3) was made. Once inside the conditioned room, the first stage of the procedure consisted of a brief series of questions for registering the subject’s data in i-CARE (sociodemographic data). This lasted for approximately 2 min. Subsequently, the second (acclimatization/body conditioning) stage took place. It consisted of having the subject sit in a comfortable position of his/her choice without speaking or moving for about 3 min, in order to allow them to adapt to the temperature inside the room and to regulate the subject’s vital signs at a basal level. At the end of the acclimatization/body conditioning stage, the smart sensor was placed on the child’s finger and the first thermographic image was taken and implemented as the baseline (phase 1 in i-CARE), according to the scheme shown in Figure 2. Stage three consisted of instructing the child on training with biofeedback and diaphragmatic breathing through the interactive visualization of their vital signs (HR and oxygenation in the blood percentage) on the i-CARE screen for a period of 10 min. Once stage three was completed, the fourth stage called imagery (training in relaxation through guided imagery) began. It consisted of guiding the subjects through imagination to calm places. This was attained using appropriate sounds and images. Subsequently, diaphragmatic breathing was performed for 10 min. The fifth and final stage consisted of a biofeedback activity that helped children relax through an interactive video game for a period of 5 min. At the end of the interaction with i-CARE, the last thermographic image was taken. It should be noted that the HR signal was acquired every 3 s throughout this protocol using the pulse-oximeter described in Section 2.1 and that the averages of the baseline and game stages were used for the study, since these were paired with the thermographic images, which, having been obtained at the beginning and end of the protocol, were called start and end, respectively. 

### 2.6. Smart Sensor

**Figure 5:**
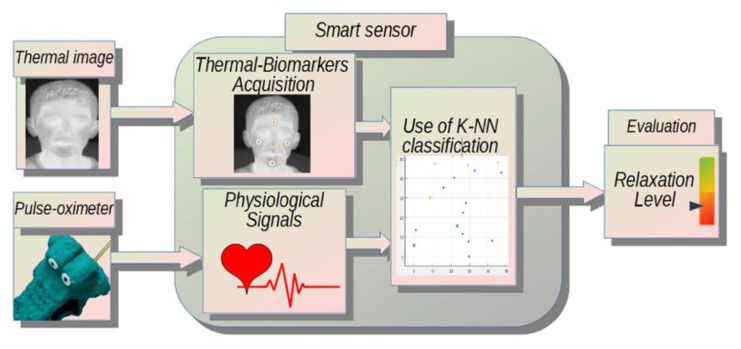
Smart sensor diagram.

Once the pertinent data had been acquired, the appropriate smart sensor was designed to be able to deliver an evaluation of the relaxation level achieved by the participant throughout the application of the protocol. Figure 5 shows a general diagram of the smart sensor.

#### 2.6.1. Thermal Image Acquisition and Processing

**Figure 6:**
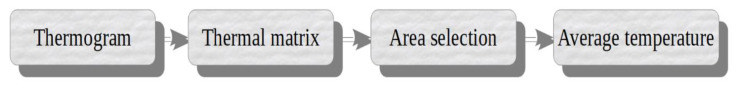
Thermal biomarker acquisition methodology.

The acquisition of thermal biomarkers followed the methodology shown in Figure 6.

The acquisition of the thermogram was carried out using the equipment described in Section 2.1 within the conditioned space mentioned in Section 2.2 and following the protocol described in Section 2.5.

To obtain the thermal matrix, Equation (1) proposed by Jadin et al. [46] was used:

(1) $$T_{r}=T_{min}+\left(\frac{T_{gray}}{T_{mgv}}\left(T_{max}-T_{min}\right)\right)$$

where $T_{r}$ is the thermal value of the pixel of the thermogram in question; $T_{min}$ and $T_{max}$ are the values of the minimum and maximum temperature in °C, respectively; $T_{gray}$ represents the gray scale value of the pixel; $T_{mgv}$ is the largest gray scale value within the thermogram.

**Figure 7:**
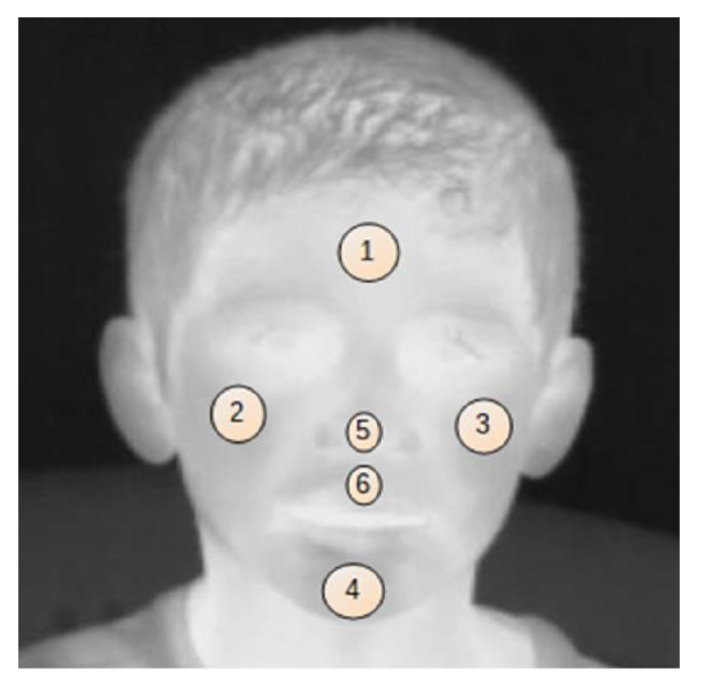
Areas of interest.

Using the thermal matrix, the areas of interest within the thermal image can be selected. For this study, these areas were as follows: (1) nose; (2) right cheek; (3) left cheek; (4) chin; (5) nose; (6) maxillary. These facial areas were associated with emotions and parasympathetic activity [16], as shown in Figure 7. The temperature of each point within these areas was calculated through the thermal matrix and an average was subsequently obtained in each section. The values obtained in this process were identified as thermal biomarkers.

There are outstanding methods that can be employed to improve the quality of thermographic images and reduce noise caused by various external sources, such as light incidents, image movement, or thermal fluctuations. One of them is the use of Principal Component Thermography (PCT) [47] or improved versions such as Candid Covariance-Free Incremental Principal Component Thermography (CCIPCT) [48,49], which allow noise to be considerably eliminated. However, for this work, it was not used because the workspace was previously conditioned to avoid any type of noise, in addition to statically taking the images.

#### 2.6.2. Acquisition and Processing of HR (Pulse)

The acquisition of HR (pulse), was performed using the pulse-oximeter described in Section 2.1. 

Data were saved throughout the application of the protocol shown in Figure 4. These data were averaged and paired with the thermographic images for the study, as shown in Section 2.5.

It is important to mention that an evaluation of HR is subject to numerous factors, including the following: the presence of pain and stress; age (HR changes with age); gender (in general, HR is higher in females); the position of the body (HR is lower in the supine position); the time of day (HR is higher in the early hours of the morning). In addition, the environmental temperature and medications (such as atropine, beta blockers, and phenylephrine) can also alter HR [50,51]. Table 1 shows the values for HR in the pediatric population; in children from 6 to 8 years old, it is equivalent to 70–135 beats per minute, whilst in children between 10 and 12 years old, it is equal to 60–120 beats per minute.

#### 2.6.3. Use of the K-NN Classifier

Once the basal and post i-CARE treatment values of each of the signals of interest (i.e., the six thermal biomarkers and HR) were obtained, their difference was calculated using Equation (2):

(2) $$\Delta_{v}=V_{f}-V_{i}$$

where $\Delta_{v}$ is the value change; $V_{f}$ is the final value, when the protocol is already finished; $V_{i}$ is the basal value. Obtaining the change in values for both the thermal biomarkers and the pulse was of vital importance for the study, since it is the change that occurs with respect to what the values were before starting the protocol.

Once the final start variation of each value had been obtained, the data was normalized using Equation (3):

(3) $$V_{n}=\frac{V_{r}-V_{min}}{V_{max}-V_{min}}$$

where $V_{n}$ is the normalized value (form 0 to 1); $V_{min}$ and $V_{max}$ represent the minimum and maximum value of the data, respectively; $V_{r}$ is the value to normalize. Normalization is really important due to the fact that the inputs for the classification have different units and homogenization is necessary for its use.

**Figure 8:**
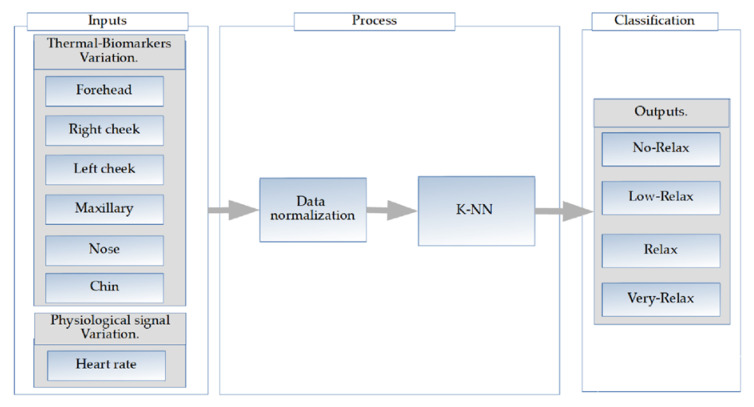
k-nearest neighbors (K-NN) inputs and outputs.

A K nearest neighbors classifier is proposed (Fine K-NN) [53], with the EUCLIDEAN distance and a number of neighbors = 1. The normalized values of the indicators (thermal biomarkers and HR variation) are used as inputs or predictors and the outputs are four possible classes, which represent the relaxation level achieved (Figure 8).

The inputs are the six thermal biomarkers and HR normalized variations that enter the classification to obtain four possible outputs or classifications, and this option obtaining process is described in Table 2.

The proposed classifications were obtained a priori when evaluating the relaxation of the subjects. This relaxation had already been obtained qualitatively with respect to the experience and knowledge of the expert in psychology and by an independent evaluation of the results of each of the participants, taking into account that when trying to decrease the anxiety and stress to induce a state of relaxation, it needs to go from being in the sympathetic to the parasympathetic system, so that the thermal biomarker temperature should gradually increase and the HR should decrease, in addition to taking into account direct observations on the change of behavior of the child when using the application. Names were assigned to the relaxation ranges according to the percentage they presented by dividing the total of 100% into four equal parts. Four possible classifications were obtained, as shown in Table 2 in the third column, where NR denotes no-relax or without relaxation, LR is low-relax or with a low level of relaxation, R is relax or with a good level of relaxation, and VR is very-relax or with a very good level of relaxation.

## 3. Results

The indicators were analyzed to determine if they were statistically significant for later use within the classifier, and this section presents the results obtained.

### 3.1. Temperatures

Thermal biomarkers were acquired according to the protocol described in Section 2.6.1. The results obtained after the analysis are shown in Table 3, where the percentage of temperature variation in the entire sample is presented.

#### Paired Two Sample for Means

To find out if the data obtained were statistically significant, they were processed using the statistical *t*-test, taking into account the start and end of each of the six thermal indicators. Table 3 displays the data obtained.

**Figure 9:**
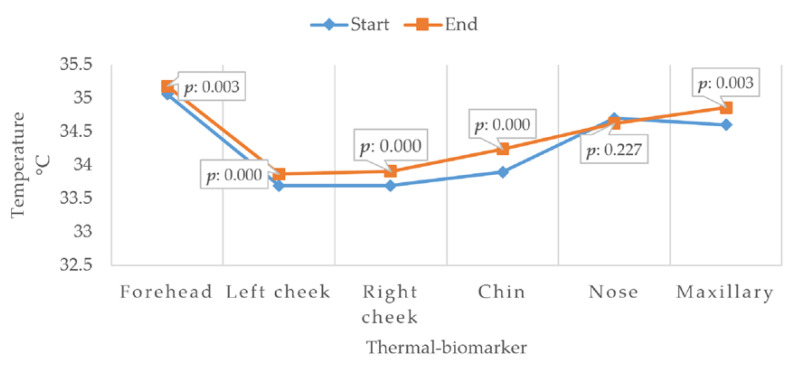
t-test: paired two sample for means (thermal biomarkers).

The graph in Figure 9 depicts a clearer appreciation of the behavior of the *p* value in the tests carried out, and the average variation in each of the indicators is plotted, highlighting the *p* value.

Only in the case of the nose was the *p* value higher than 0.05, so this indicator was discarded for the study; the rest of the values were less than 0.05, making them useful and reliable. 

**Figure 10:**
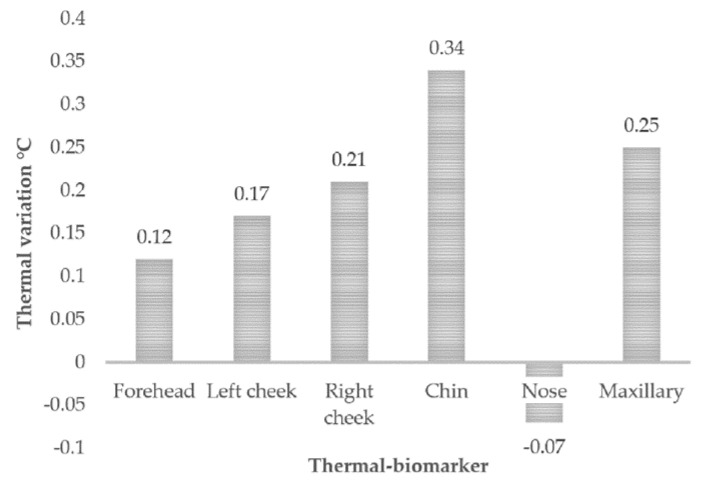
Thermal variation in the six thermal biomarkers.

It can be seen from Figure 9 and Table 2 that the average temperature in thermal biomarkers varied similarly in each of the cases, with the exception of the nose, which is also the one that presented a lower *p* value, being the only one to not present statistical significance. Figure 10 shows a clearer representation of the temperature changes that occurred in each of the areas of interest.

A clear trend towards an increasing temperature can be observed in the thermal biomarkers, with the chin showing the greatest average change, followed by the maxillary muscle and the two cheeks, and then the forehead with the lowest increase. The nose, as previously mentioned, was discarded due to a lack of statistical significance. 

### 3.2. Pulse

The HR was acquired throughout the study, but as mentioned in Section 2.6, only the values of the first and last sections were used because these values were paired with the temperature values. The variation column in Table 4 shows the average variation in these two indicators.

#### Paired Two Sample for Means

As in the case of temperature, the data were processed using the *t*-test to find statistical significance. Table 4 shows the results obtained.

**Figure 11:**
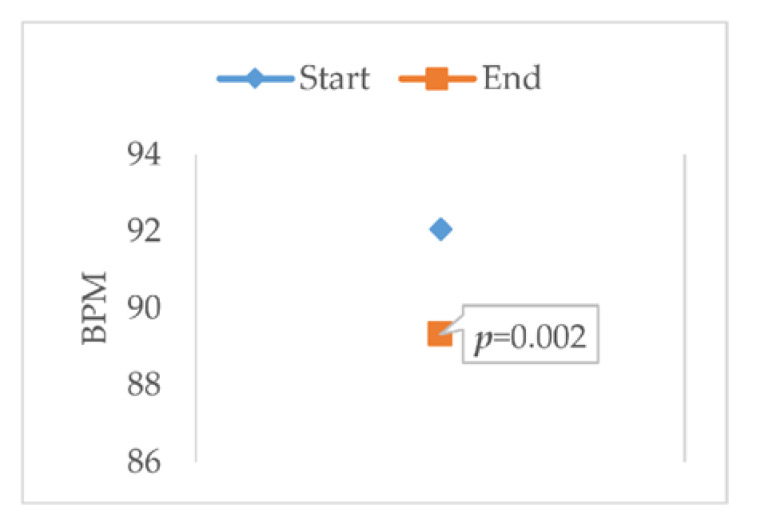
t-test: paired two sample for means of the pulse indicator (BPM: beats per minute).

It can be seen that, on average, the pulse decreased considerably. The graph in Figure 11 illustrates a better appreciation of the behavior of the *p* value in the tests carried out, and the average variation in each of the indicators is plotted, highlighting the *p* value.

The *p* value obtained for HR (pulse) is less than 0.05, so it is useful for this study and was used as an input or indicator for the classifier.

### 3.3. K-NN Classification

This section presents the performance of the proposed K-NN classifier, validated by the K-fold cross validation method. For the implementation and validation of the classifier, the MATLAB software was used [53].

#### K-Fold Cross Validation

**Figure 12:**
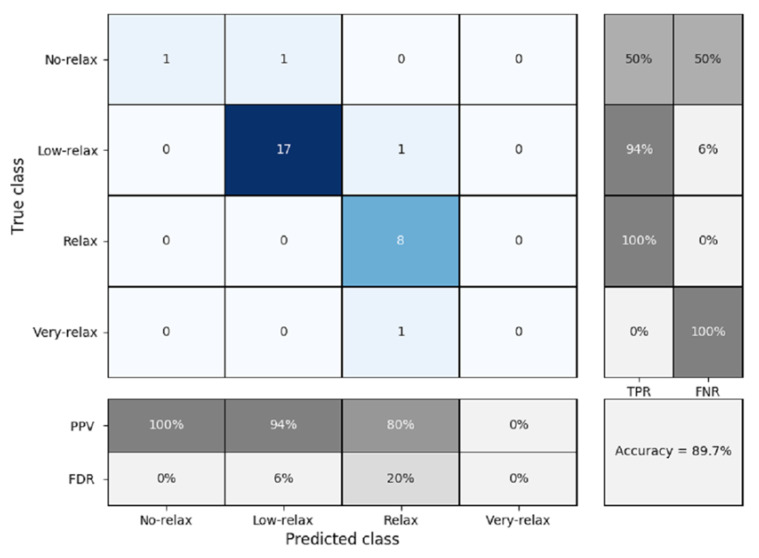
Confusion matrix for k-nearest neighbors (K-NN) classification and five-fold cross validation (all participants), where PPV = positive predicted value, FDR = false discovery rate, TPR = true positive rate or sensitivity, and FNR = false negative rate or specificity.

To validate this study, K-fold cross validation was used with a K = 5 due to the number of samples available. Figure 12 shows the confusion matrix obtained.

**Figure 13:**
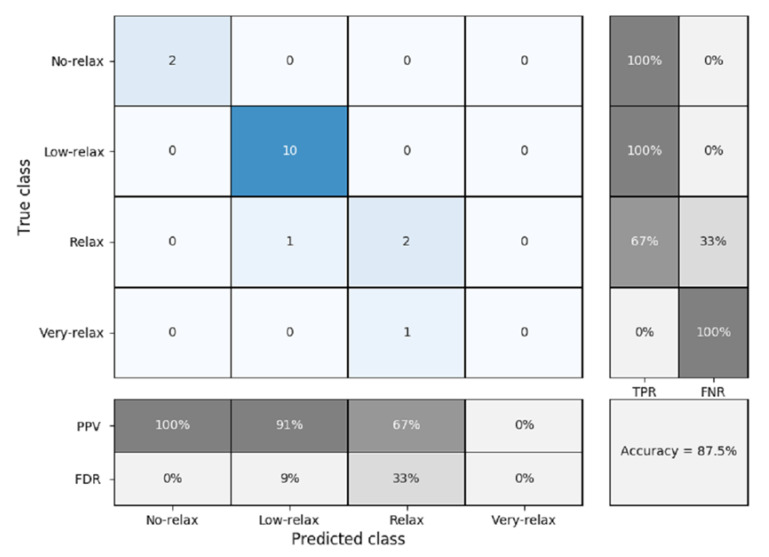
Confusion matrix for k-nearest neighbors (K-NN) classification and five-fold cross validation (boys), where PPV = positive predicted value, FDR = false discovery rate, TPR = true positive rate or sensitivity, and FNR = false negative rate or specificity.

The percentage of accuracy obtained was 89.7%, with prediction failures mainly occurring in the most remote areas, which are no-relax and very-relax. The best results were found in the case of the relax and low-relax classifier, because in these two areas, there were many more tests for training. It can be seen that the true positive rates are very high in the center of the classifier, but they fall on the outskirts. This can be seen in the same way for the percentage of values correctly and incorrectly predicted. The values of the true positive rate or specificity are high in the case of the relax and low-relax classes, with values of 100% and 94%, respectively, providing good pressure to the classifier, since these are the classes where most of the results were found. On the other hand, the no-relax and very-relax classifiers had high values of the false discovery rate or specificity, of 50% and 100%, respectively. Lastly, the positive predicted value was acceptable, being greater than or equal to 80%, with the exception of the very-relax classifier, in which there was no prediction. Boy and girl participants were evaluated independently to find out if there was any difference between them. Figure 13 shows the confusion matrix obtained for the case of boys.

**Figure 14:**
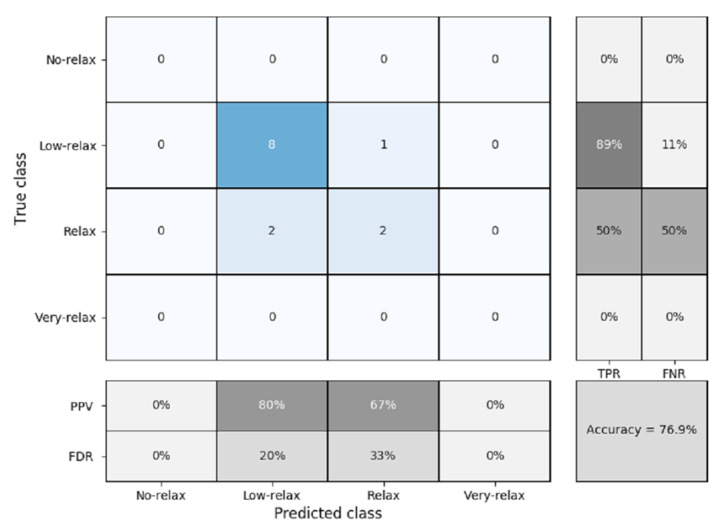
Confusion matrix for k-nearest neighbors (K-NN) classification and five-fold cross validation (girls), where PPV = positive predicted value, FDR = false discovery rate, TPR = true positive rate or sensitivity, and FNR = false negative rate or specificity.

As can be seen in Figure 13, the precision obtained for male only is less than when boys and girls are considered together (Figure 12), but still maintains a good accuracy of 87.5%. The sensitivity, specificity, positive predicted value, and false discovery rate remained similar to when the two genders were grouped together. Figure 14 shows the confusion matrix for when only the girls were measured. In this case, the precision is reduced to 76.9%. As shown in the figure, numeric values were only found in the sections at the center of the matrix (i.e., low-relax and relax). This is due to the fact that no values were registered for girls with the ratings of no-relax or very-relax.

**Figure 15:**
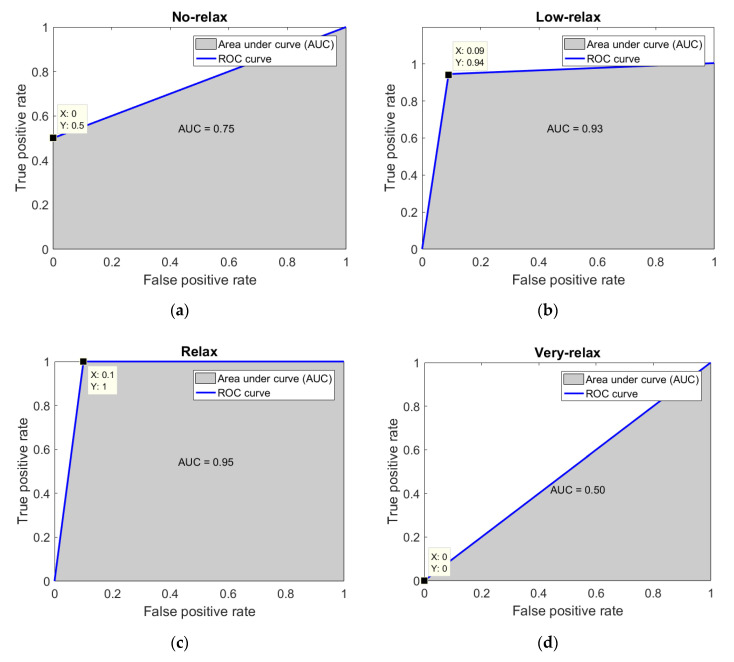
Receiver operating characteristic (ROC) curves of classifier activity: (**a**) no-relax; (**b**) low-relax; (**c**) relax; (**d**) very-relax.

To achieve a better understanding of the behavior of the proposed classifiers, receiver operating characteristic (ROC) curves were constructed. These are shown in Figure 15. The results displayed in these figures corroborate the findings attained by the confusion matrix analysis (Figure 12, Figure 13 and Figure 14). The data point shown in each of the graphs represents the location of the current classifier value for each level of relaxation achieved. It can be seen that, in the very-relax case, the classification value is very low. This results from the fact that it was decided that the probability of obtaining this classification was very low due to the small number of samples which fell within this condition. In addition, priority was given to testing the hypothesis that exposure to i-CARE treatment induced relaxation in the children. The results clearly show that the low-relax and relax options had the largest areas under the curve (AUC), with values over 0.9 in both cases and equal to 0.75 in the case of no-relax. This is consistent with the hypothesis that exposure to i-CARE induces significant relaxation in the children treated.

After analyzing the results obtained, it became evident that at the end of the process, the temperature values increased and the HR values decreased, and the classifier showed a good accuracy = 89.7%, with a difference between the evaluation of boys and girls.

## 4. Discussion

In this work, two main objectives were sought: (1) to assess the effectiveness of the i-CARE application to induce relaxation in vulnerable children, and (2) to validate the use of a novel non-invasive smart sensor technique to evaluate relaxation levels in children. The smart sensor system was based on biofeedback and thermal and HR measurements. To obtain the degree of relaxation, a K-NN classifier was used. This was accomplished by processing the input of the thermal biomarkers and HR measurements. This information was processed to yield four possible levels of relaxation: no-relax, low-relax, relax, and very-relax. The evaluation of the smart sensor yielded a very high accuracy of 89.7%. This value is at least as high as that reported by other investigators (i.e., 85.17% [54,55,56]). In our study, the classifiers which provided the best resolution (signal/noise) were the relax and low-relax states. This is significant because these were the main levels reached by the majority of children after exposure to the i-CARE treatment.

In an attempt to assess the effect of experimental manipulation (i-CARE administration) on stress and anxiety levels in children from out-of-home care, we measured two physiological parameters before and after exposure to the i-CARE protocol. These parameters were the (i) temperature of the facial skin in six different locations (thermal biomarker) and (ii) HR (physiological marker) [57]. Our results show that the administration of the i-CARE protocol produced (1) significant increases in thermal measurements at five of the six face locations. This result is easily interpreted by suggesting that the protocol induced a significant increase in parasympathetic activity [16]. Additionally, there was (2) a clear tendency to reduce HR. The trend was consistent with increasing relaxation [57].

There are a number of applications or technology-assisted approaches that can be employed to reduce anxiety and stress or induce relaxation in children and young people which use biofeedback techniques [58,59], yet there is no reliable way to evaluate the functioning of these approaches, since there is no method or layered software available to evaluate them. This work offers the possibility of implementing both the relaxation process by means of i-CARE and the acquisition of physiological variables of anxiety based on the BAN paradigm, in order to evaluate the level of relaxation achieved by children.

Numerous published studies have presented the classification of emotions, including the separation of positive and negative emotions [60,61], but to the best of our knowledge, there are no classifiers that provide a gradual classification of the level of relaxation, especially in a specific population such as a group of children in out-of-home care.

The tests carried out for the training of this smart sensor were carried out in the field, without removing the study subjects from their comfort area, although all of the tests were carried out in a controlled space to improve the quality of the thermographic images. This area was a space adapted and equipped within the same care home facility where the children were housed. This arrangement allowed the participants not to be disturbed by external factors.

A large part of the equipment that has been used for data acquisition in previous studies has been invasive, since the signals used for the evaluation have been EEG, ECG, or EMG [54,60,62]. The nature of these invasive procedures may disturb the very parameter to be measured. In the case of the system described in this paper, all of the equipment used is non-invasive, portable, and readily available.

In order to improve the accuracy of this sensor, additional studies should be carried out to assess the efficacy of novel and old classifiers. In addition, further research should aim to understand why the precision is higher in boys than girls and explore the possibility of adding new indicators from the sensing of vital signs, such as oxygen saturation in the blood and the blood pressure.

## 5. Conclusions

The present work had two main goals: (1) to validate the use of a novel non-invasive smart sensor based on infrared measurements of facial temperature and HR to evaluate the relaxation level in children, and (2) to assess the hypothesis that an innovative application called i-CARE, based on relaxation and biofeedback techniques, could be used to induce relaxation in vulnerable, out-of-home children. A study was carried out to obtain the significant biomarkers that indicate the presence of relaxation, which are the infrared temperature measurements made at the forehead, right and left cheek, chin, and maxilla; the HR was measured with a custom-made finger sensor. Quantification of the relaxation level was accomplished using a K-NN classifier, which provided four possible classifications at the end of this process: no-relax, low-relax, relax, and very-relax. The accuracy of the smart sensor was 89.7%. The results demonstrate that the i-CARE method is effective in inducing relaxation in out-of-home children, and that the smart sensor used provides reliable measurements of relaxation levels in children exposed to out-of-home care conditions, with better results in boys than girls. Access to the population addressed in this work is very complicated due to various political, economic, and social factors, but it is these vulnerable populations that most need to be taken into account for future research since they are the people who most require our help.

## Figures and Tables

**Table 1:** Normative ranges of the heart rate in the pediatric population.

Age	Weight (kg)	Heart Rate (BPM) ^1^
6 years	20	70–135
8 years	25	70–135
10 years	30	60–120
12 years	40	60–120

^1^ Where BPM means beats per minute (adapted from [52]).

**Table 2:** Proposed classification.

Number	Name	Percentage ^1^
1	No-relax	0% ≤ NR ≤ 25%
2	Low-relax	25% < LR ≤ 50%
3	Relax	50% < R ≤ 75%
4	Very-relax	75% < NR ≤ 100%

^1^ Where the values of the percentage were obtained from the normalized values: 0–1 to 0–100%.

**Table 3:** *t*-test: paired two sample for means (thermography).

Thermal Biomarker	$M_{s}{\;}^{1}$	$M_{e}{\;}^{2}$	*P* ^3^	Δ*T* ^4^
Forehead	35.05	35.17	0.003	0.12
Left cheek	33.69	33.86	0.000	0.17
Right cheek	33.69	33.90	0.000	0.21
Chin	33.89	34.23	0.000	0.34
Nose	34.69	34.62	0.227	−0.07
Maxillary	34.60	34.85	0.003	0.25

^1^ Where $M_{s}$ is the initial mean, ^2^ $M_{e}$ is the final mean, ^3^ *P* is the significance, and ^4^ Δ*T* is the temperature increase between the final mean and the initial mean.

**Table 4:** *t*-test: paired two sample for means (pulse).

Indicator	$M_{s}{\;}^{1}$	$M_{e}{\;}^{2}$	*P* ^3^	Δ*I* ^4^
Pulse	92.08	89.31	0.002	−3.49

^1^ Where $M_{s}$ is the initial mean, ^2^ $M_{e}$ is the final mean, ^3^ *p* is the significance, and ^4^ Δ*I* is the increment of the indicator between the final mean and the initial mean.
